# Carnitine transporter OCTN2 and carnitine uptake in bovine kidney cells is regulated by peroxisome proliferator-activated receptor β/δ

**DOI:** 10.1186/1751-0147-56-21

**Published:** 2014-04-09

**Authors:** Xiaodan Zhou, Robert Ringseis, Gaiping Wen, Klaus Eder

**Affiliations:** 1 Institute of Animal Nutrition and Nutritional Physiology, Justus-Liebig-University, Heinrich-Buff-Ring 26-32, 35392 Giessen, Germany

**Keywords:** Bovine kidney cell, Novel organic cation transporter 2, Peroxisome proliferator-activated receptor β/δ

## Abstract

**Background:**

Peroxisome proliferator-activated receptor α (PPARα), a central regulator of fatty acid catabolism, has recently been shown to be a transcriptional regulator of the gene encoding the carnitine transporter novel organic cation transporter 2 (*OCTN2*) in cattle. Whether PPARβ/δ, another PPAR subtype, which has partially overlapping functions as PPARα and is known to share a large set of common target genes with PPARα, also regulates *OCTN2* and carnitine transport in cattle is currently unknown. To close this gap of knowledge, we studied the effect of the PPARβ/δ activator GW0742 on mRNA and protein levels of *OCTN2* and carnitine uptake in the presence and absence of the PPARβ/δ antagonist GSK3787 in the bovine Madin-Darby bovine kidney (MDBK) cell line.

**Findings:**

Treatment of MDBK cells with GW0742 caused a strong increase in the mRNA level of the known bovine PPARβ/δ target gene *CPT1A* in MDBK cells indicating activation of PPARβ/δ. The mRNA and protein level of *OCTN2* was clearly elevated in MDBK cells treated with GW0742, but the stimulatory effect of GW0742 on mRNA and protein level of *OCTN2* was completely blocked by GSK3787. In addition, GW0742 increased Na^+^-dependent carnitine uptake, which is mediated by OCTN2, into MDBK cells, whereas treatment of cells with the PPARβ/δ antagonist completely abolished the stimulatory effect of GW0742 on carnitine uptake.

**Conclusions:**

The present study shows for the first time that gene expression of the carnitine transporter OCTN2 and carnitine transport are regulated by PPARβ/δ in bovine cells. These novel findings extend the knowledge about the molecular regulation of the *OCTN2* gene and carnitine transport in cattle and indicate that regulation of *OCTN2* gene expression and carnitine transport is not restricted to the PPARα subtype.

## Findings

The peroxisome proliferator-activated receptors (PPARs) are ligand-activated transcription factors which play important roles in many metabolic and regulatory pathways through regulating the expression of a large set of target genes [1]. The ligands of PPARs include fatty acids and fatty acid derivatives and are therefore designated as transcriptional sensors of fatty acids [2]. In addition, synthetic compounds like the fibrate class of lipid lowering drugs or the insulin-sensitizing thiazolidinediones are well documented ligands of PPARs. From the PPARs, three different subtypes exist which have isotype-specific but also partially overlapping functions. For instance, both, the PPARα and the PPARβ/δ subtype are central regulators of fatty acid catabolism since both subtypes control the expression of genes encoding proteins involved in cellular fatty acid uptake, intracellular fatty acid transport, mitochondrial fatty acid uptake, and mitochondrial and peroxisomal fatty acid oxidation [1,3,4]. Recent studies convincingly demonstrated that PPARα is also a key regulator of genes involved in carnitine transport like novel organic cation transporter 2 (*OCTN2*) and carnitine synthesis like γ-butyrobetain dioxygenase (*BBD*) in many species including dairy cattle [5-8]. Activation of PPARα in dairy cattle occurs physiologically during early lactation due to extensive mobilization and release of fatty acids from adipose tissues which are taken up into the liver and non-hepatic tissues and bind to and activate PPARα [9,10]. The above mentioned PPARα dependence of OCTN2 and BBD expression provides a plausible explanation for the recent finding that OCTN2 and BBD in the liver are strongly up-regulated during early lactation in high-producing dairy cows [11]. However, whether PPARβ/δ also regulates genes involved in carnitine homeostasis in cattle is currently unknown. PPARα and PPARβ/δ share a large set of common target genes involved in fatty acid catabolism. In addition, carnitine transport and synthesis are intrinsically linked to fatty acid catabolism, because fatty acid transport into the mitochondrial matrix is carnitine-dependent [12]. Thus, we hypothesized that PPARβ/δ also regulates genes involved in carnitine homeostasis in cattle. To verify our hypothesis we studied the effect of the PPARβ/δ activator GW0742 on mRNA and protein levels of *OCTN2* and carnitine transport in a bovine kidney cell line. Using this cell line we have very recently shown that *OCTN2* gene expression and carnitine transport are stimulated by a PPARα agonist [13]. However, kidney cells are also of relevance to study the effect of PPARβ/δ agonists in this regard because PPARβ/δ is known to be highly expressed in the kidney and OCTN2-mediated carnitine transport represents the transport mechanism for tubular reabsorption of carnitine in the kidney and is therefore fundamental for maintaining normal carnitine levels in serum [14]. We did not consider the effect of GW0742 on genes involved in carnitine synthesis in this cell line, because the kidney, unlike the liver, is not capable of synthesizing carnitine due to the lack of BBD.

**Figure 1:**
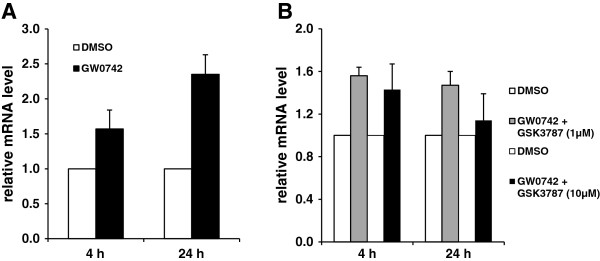
**Effect of incubation time and effect of concentration of PPARβ/δ antagonist on relative mRNA level of OCTN2 in MDBK cells. A**, Effect of treatment with 1 μM of PPARβ/δ agonist GW0742 for 4 and 24 h on mRNA level of *OCTN2*. **B**, Effect of treatment with 1 and 10 μM of PPARβ/δ selective antagonist GSK3787 for 4 and 24 h on mRNA level of *OCTN2.* Bars represent means ± SD of three independent experiments and are expressed as fold of DMSO-treated control cells.

Madin-Darby bovine kidney (MDBK) cells obtained from Cell Lines Service (Eppelheim, Germany) were cultivated in HyClone Minimum Essential Media/Earle’s Balanced Salt Solution (MEM/EBSS) medium supplemented with 10% FBS and 0.05 mg/mL gentamicin (all from Invitrogen, Karlsruhe, Germany) at 37°C in a humidified atmosphere of 95% air and 5% CO_2_[13]. After reaching 80% confluence, MDBK cells were treated with 1 μM of the PPARβ/δ selective agonist GW0742 (Sigma-Aldrich, Steinheim, Germany) [dissolved in dimethylsulfoxide (DMSO); both from Sigma-Aldrich, Steinheim, Germany] in MEM/EBSS medium without FBS but 5 mg/L bovine insulin (Sigma-Aldrich, Steinheim, Germany) for 24 h. The incubation concentration of GW0742 was chosen based on published literature [15], in which treatment with 1 μM resulted in strong activation of PPARβ/δ. The incubation time was selected based on results from initial time course experiments (4 h, 24 h) demonstrating that the effect of GW0742 was stronger at 24 h (Figure 1A). Cells treated with vehicle alone (DMSO) were used as control. Incubation media of control cells contained the same vehicle concentration of 0.1% (v/v). For experiments using a PPARβ/δ inhibitor, cells were co-treated with 10 μM of the PPARβ/δ selective antagonist GSK3787 (Sigma-Aldrich) for 24 h. The incubation time and incubation concentration of GSK3787 was chosen also based on results from initial titration and time course experiments (concentration: 1 and 10 μM; time: 4 h, 24 h) demonstrating that inhibition of the agonist effect by GSK3787 was strongest at 10 μM and 24 h (Figure 1B). Following incubation, media was aspirated, the cell layer was washed once with phosphate-buffered saline, and plates including the attached cells were immediately stored at −80°C. All incubations were run in triplicate and each experiment was repeated three times. The mRNA levels of genes of interest (reference and target genes) in MDBK cells were determined by means of qPCR. Prior to qPCR, RNA from cells was isolated by adding TrizolTM reagent (Invitrogen, Karlsruhe, Germany) directly into the wells, and pipetting the lysed cells up and down 2–3 times. cDNA was synthesized in less than a week after RNA extraction from 1.2 μg of total RNA using 100 pmol dT18 primer (Eurofins MWG Operon, Ebersberg, Germany), 1.25 μL 10 mmol/L dNTP mix (GeneCraft, Lüdinghausen, Germany), 5 μL buffer (Fermentas, St. Leon-Rot, Deutschland), and 60 units M-MuLV Reverse Transcriptase (MBI Fermentas, St. Leon-Rot, Germany) at 42°C for 60 min, and a final inactivating step at 70°C for 10 min in a Thermal Cycler. qPCR was performed using 2 μL cDNA combined with 18 μL of a mixture composed of 10 μL KAPA SYBR FAST qPCR Universal Mastermix (Peqlab, Erlangen, Germany), 0.4 μL each of 10 μM forward and reverse primers and 7.2 μL DNase/RNase free water in 0.1 mL tubes (Ltf Labortechnik, Wasserburg, Germany). Ct-values of target genes and reference genes were obtained using Rotorgene Software 5.0 (Corbett Research). For determination of relative expression levels relative quantities were calculated using GeNorm normalization factor according to Vandesompele *et al*. [16]. The normalization factor was calculated as the geometric mean of expression data of the three most stable (*ACTB*, *ATP5B*, *SDHA*) out of five tested potential reference genes (*ACTB*, *ATP5B*, *PPIA*, *RPS9*, *SDHA*). Primer characteristics and qPCR performance data for the reference genes and target genes have been published recently [9]. Means and SD were calculated from normalized expression data for samples of the same treatment group. The mean of the vehicle (DMSO) control group was set to 1 and mean and SD of the GW0742 and GW0742 + GSK3787 groups were scaled proportionally. For immunoblot analysis, MDBK cells were lysed with RIPA lysis buffer (50 mM Tris, pH 7.5; 150 mM NaCl, 1 mM EDTA, 1% Triton X-100, 0.1% SDS, 1% sodium deoxycholate) containing protease inhibitors (Sigma-Aldrich). Following determination of protein concentration of the cell lysates, 25 μg protein from the cell lysates were separated on a 10% SDS-PAGE, and proteins were transferred to a nitrocellulose membrane. Subsequently, membranes were blocked overnight at 4°C in blocking solution (5% non-fat dried milk powder), and then incubated with antibodies against OCTN2 (polyclonal anti-OCTN2 antibody; dilution 1:500; Abcam, Cambridge, UK) and β-actin (monoclonal anti-β-actin; dilution 1:500, Abcam, Cambridge, UK) overnight at 4°C and for 2 h at RT, respectively. Following a washing step, the membranes were incubated with a horseradish peroxidase conjugated secondary monoclonal anti-mouse-IgG antibody (1:5000, Jackson Immuno Research, Suffolk, UK) for 1 h at room temperature. Finally, the blots were developed by using the Amersham^TM^ ECL Plus Western Blotting Detection System (GE Healthcare, Munich, Germany) and detected by a chemiluminescence imager (Syngene, Cambridge, UK). The signal intensities of specific bands were quantified using GeneTools software (Syngene, Cambridge, UK). Carnitine uptake experiments in MDBK cells using methyl-L-[^3^H]-carnitine (80 mCi/mmol; American Radiolabeled Chemicals, St. Louis, USA) were performed as described recently in detail [13]. To study the Na^+^ dependence of carnitine uptake, the incubation buffer contained either 0, 25 or 125 mM NaCl and 4.8 mM KCl, 5.6 mM D-glucose, 1.2 mM CaCl_2_, 1.2 mM KH_2_PO_4_, 1.2 mM MgSO_4_, and 5 mM HEPES. Radioactivity in cell lysates determined by scintillation counting (Perkin Elmer Liquid Scintillation Analyzer Tri-Carb 2900TR, Rodgau, Germany) was related to protein content of cell lysates as determined by the bicinchoninic acid protein assay with BSA as standard. Carnitine uptake is expressed as the amount of L-[^3^H]-carnitine taken up per mg cell protein within 30 min. Statistical evaluation of treatment effects was carried out by one-way ANOVA and Duncan’s multiple range test.

**Figure 2:**
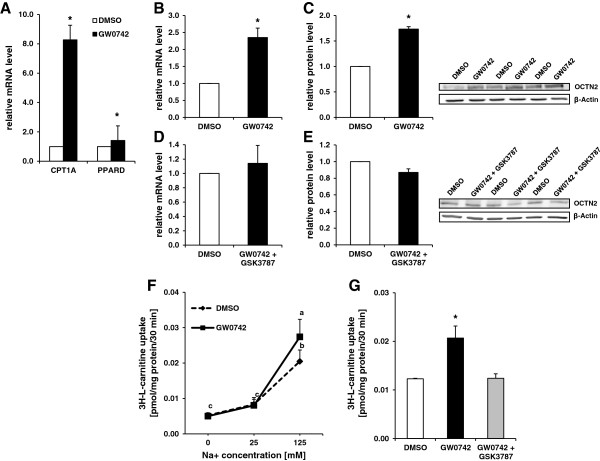
**The carnitine transporter OCTN2 and carnitine uptake in MDBK cells is regulated by PPARβ/δ. A**, Effect of treatment with 1 μM of GW0742 for 24 h on mRNA levels of *CPT1A* and *PPARD*. **B-E**, Effect of treatment with 1 μM of GW0742 for 24 h in the absence **(B, C)** and presence **(D, E)** of PPARβ/δ selective antagonist GSK3787 (10 μM) on relative mRNA **(B, D)** and protein levels **(C and E)** of *OCTN2*. **A**, **B** and **D**, Bars represent means ± SD of three independent experiments and are expressed as fold of DMSO-treated control cells. **C** and **E**, Bars represent data from densitometric analysis and are means ± SD of three independent experiments. Immunoblots specific to OCTN2 and β-Actin as internal control are shown for one independent experiment; immunoblots for the other experiments revealed similar results. Data represent means ± SD of three independent experiments and are expressed as fold of DMSO-treated control cells. *Different from DMSO-treated control, *P <* 0.05. **F** and **G**, Effect of treatment with 1 μM of GW0742 on uptake of L-[^3^H]-carnitine (10 nM, specific radioactivity 80 Ci/mmol). F, Uptake of L-[^3^H]-carnitine by MDBK cells treated for 24 h with either GW0742 or DMSO (control) at different Na^+^ concentrations (0, 25 and 125 mM NaCl) was studied over 30 min. **G**, Uptake of L-[^3^H]-carnitine by MDBK cells treated for 24 h with either GW0742, GW0742 together with GSK3787 (10 mM) or DMSO (control) at 125 mM NaCl was studied over 30 min. Data represent means ± SD of three independent experiments each performed in triplicate. ^a,b,c^Data with different superscript letters differ, *P <* 0.05. *Different from DMSO-treated control, *P <* 0.05.

In the first step, we investigated whether treatment of MDBK cells with the PPARβ/δ agonist GW0742 (1 μM) causes activation of PPARβ/δ. Activation of PPARβ/δ was evidenced by strongly increased mRNA levels of the known bovine PPARβ/δ target gene *CPT1A* in MDBK cells treated with GW0742 (*P* < 0.05; Figure 2A). The mRNA level of *PPARβ/δ* was slightly elevated by treatment with GW0742 (*P* < 0.05; Figure 2A). Next, we studied the effect of GW0742 on the mRNA level of *OCTN2* in MDBK cells. As shown in Figure 2B, the mRNA level of *OCTN2* was clearly elevated in MDBK cells treated with GW0742 (*P* < 0.05) indicating that bovine *OCTN2* gene transcription is regulated by PPARβ/δ. In addition, induction of the *OCTN2* gene by GW0742 in MDBK cells was also observed at the protein level (*P* < 0.05; Figure 2C). To further confirm the PPARβ/δ dependence of this effect, we studied the effect of GW0742 in the presence of GSK3787 (10 μM) on *OCTN2* gene expression. GSK3787 is a newly identified PPARβ/δ antagonist that can irreversibly attenuate the activity of PPARβ/δ by forming a covalent bond with a cysteine residue in the ligand binding domain of PPARβ/δ [17]. As illustrated in Figure 2D and E, the stimulatory effect of GW0742 on mRNA and protein levels of *OCTN2* in MDBK cells was completely blocked by the PPARβ/δ antagonist indicating that *OCTN2* gene expression is regulated by PPARβ/δ in bovine kidney cells. In a further step, we studied whether up-regulation of OCTN2 by GW0742 leads to an increased carnitine uptake. For this, we determined the uptake of methyl-L-[^3^H]-carnitine into MDBK cells incubated with or without GW0742 at different NaCl concentrations in the incubation medium. As shown in Figure 2F, GW0742 increased carnitine uptake into MDBK cells at a NaCl concentration of 125 mM in the incubation media (*P* < 0.05) but not at 0 and 25 mM NaCl. This indicated that the PPARβ/δ agonist stimulates specifically the OCTN2-mediated carnitine uptake which is known to be sodium-dependent [18]. Finally, we provided clear evidence for the PPARβ/δ dependence of the GW0742-induced increase of carnitine uptake in showing that treatment of MDBK cells with the PPARβ/δ antagonist completely abolished the stimulatory effect of GW0742 on carnitine uptake (Figure 2G). In summary, these novel findings extend the knowledge about the molecular regulation of the OCTN2 gene and carnitine transport which have been convincingly demonstrated to be regulated by PPARα in cattle but also in several other species [6]. The fact that OCTN2 gene expression and carnitine transport are obviously regulated by both, PPARα and PPARβ/δ, is not surprising given that these two PPAR subtypes have partially overlapping functions. Namely, both PPAR subtypes play important roles in the regulation of mitochondrial fatty acid oxidation, which is dependent on the presence of carnitine, and therefore share a large set of common target genes involved in fatty acid oxidation [15]. Thus, our recent observation in high-producing dairy cows that OCTN2 is strongly up-regulated in the liver during early lactation [9] might be mediated by the activation of both PPAR subtypes because the fatty acids released from adipose tissue during this state are ligands and activators of both PPAR subtypes.

## Conclusions

The present study shows for the first time that gene expression of the carnitine transporter OCTN2 and carnitine transport are regulated not only by PPARα but also by PPARβ/δ in bovine cells.

## Competing interests

The authors declare that they have no competing interests.

## Authors’ contributions

XZ conducted the cell culture experiments, performed PCR analyses, immunoblot analyses, uptake experiments and statistical analyses and wrote the manuscript. GW supervised PCR analyses and helped to draft the manuscript. RR participated in the design and coordination of the study and helped to draft the manuscript. KE conceived of the study, participated in its design and coordination and helped to draft the manuscript. All authors read and approved the final manuscript.
